# Utilization of OnCloud© to determine educational needs of the clinical perfusion student

**DOI:** 10.1186/1749-8090-8-S1-O256

**Published:** 2013-09-11

**Authors:** B Forsberg

**Affiliations:** 1 International Children's Heart Foundation, Memphis, TN, USA; 2 Cardiovascular Perfusion Department, College of Health, Barry University, Miami Shores, FL, USA

## Background

The American Society of Extracorporeal Technology (AmSECT) has distributed a recommendation standard of practice and essential guidelines based on clinical evidence and currently accepted perfusion practice worldwide. These are integrated as learning objectives for clinical perfusion education. OnCloud©, an informatics and quality management program has been developed to collect and quantify procedural data to accurately produce quality control reports of perfusion practice incorporating these standards of practice.

## Methods

Fifty pediatric congenital heart surgery cases involving cardiopulmonary bypass (CPB) where a student was the primary perfusionist were retrospectively entered into the OnCloud© database for quality control analysis. Thirty integrated OnCloud© quality control indicators with predetermined threshold percentages were employed. When a threshold percentage was not achieved it was determined that further education was necessary to improve mastery of the learning objective correlating to the failed threshold of the quality indicator.

## Results

The OnCloud© quality control report reveled that six of the thirty quality indicators failed to meet the minimum thresholds. The report provided the indicator, the student achieved percentage, and the established threshold percentage. These included; pCO_2 35-45 mmhg (56.6%/85%), Hematocrit > 30% (86.5%/90%), Charting at 15 minute intervals (27.3%/99%), checklist completed (24.5%/99%), patient weaned from CPB (92%/99%), and procedure terminated without incident (94%/100%).

## Conclusion

Due to the dynamic learning environment perfusion education entails, course corrections must be undertaken to ensure mastery of critical aspects of the conduct of cardiopulmonary bypass. By using this software during clinical activity performed by the perfusion student, we can accurately assess deficiencies in clinical performance using evidence-based criteria and formulate a proper curriculum to ensure mastery.

